# Ethnic differences in the association between blood pressure components and chronic kidney disease in middle aged and older Asian adults

**DOI:** 10.1186/1471-2369-14-86

**Published:** 2013-04-17

**Authors:** Charumathi Sabanayagam, Boon Wee Teo, E Shyong Tai, Tazeen H Jafar, Tien Yin Wong

**Affiliations:** 1 Singapore Eye Research Institute, 11 Third Hospital Avenue, #06- 13, SNEC Bldg, Singapore, Singapore; 2 Office of Clinical Sciences, Duke-NUS Graduate Medical School, Singapore, Singapore; 3 Department of Ophthalmology, Yong Loo Lin School of Medicine, National University of Singapore, Singapore, Singapore; 4 Department of Medicine, Yong Loo Lin School of Medicine, National University of Singapore, Singapore, Singapore; 5 Program in Health Services & Systems Research, Duke-NUS Graduate Medical School, Singapore, Singapore

**Keywords:** Asians, Blood pressure, Chronic kidney disease, Glomerular filtration rate, Singapore

## Abstract

**Background:**

Chronic kidney disease (CKD) is an emerging public health problem worldwide. Previous studies have shown an association between blood pressure (BP) and CKD. However, it is not clear if there are ethnic differences in this association. We examined the association between BP and CKD in a multi-ethnic Asian population in Singapore.

**Methods:**

We analysed data from three large population-based studies conducted between 2004–2011, (n=3,167 Chinese, 3,082 Malays and 3,228 Indians) aged 40–80 years. CKD was defined as an estimated glomerular filtration rate <60 mL/min/1.73m^2^ from serum creatinine. Hypertension was defined as a self-reported current use of antihypertensive medication or systolic BP ≥140 mm Hg or diastolic BP ≥90 mm Hg. We also analysed the association of CKD with individual BP components.

**Results:**

The prevalence of both hypertension and CKD was higher among Malays (68.6, 21%) compared to Chinese (57.9, 5.9%) and Indians (56.0, 7.4%), but treatment for hypertension was lower among Malays (53.4%) compared to Chinese (89.8%) and Indians (83.1%). Hypertension was associated with CKD in all three ethnic groups (OR [95% CI] = 2.71 [1.59-4.63], 2.08 [1.62-2.68], 2.43 [1.66-3.57] in Chinese, Malays and Indians). Among the BP components, both systolic and diastolic BP were associated with CKD in Malays whereas, systolic BP was not significantly associated with CKD, and diastolic BP showed an inverse association which was explained by anti-hypertensive medication use in Chinese and Indians.

**Conclusions:**

Hypertension was associated with CKD in Chinese, Malays and Indians. However, the BP components were associated with CKD only in Malays.

## Background

Hypertension is an established risk factor for chronic kidney disease (CKD) associated with increased risk for progression to renal failure and adverse cardiovascular outcomes [1-3]. Evidence from clinical trials suggest a beneficial effect of optimal BP control on CKD progression [4,5]. Hypertension has been shown to be an important risk factor for CKD in addition to diabetes in Western populations [6,7]. Recent studies conducted in the US have shown that hypertension of all ranges including prehypertension [7,8] and all components including systolic BP [8-13], diastolic BP [11] and pulse pressure [13] are associated with CKD. Ethnic minorities including blacks and Asian Americans [14] were shown to be at increased risk of developing CKD compared to whites in the US. The prevalence of both hypertension and CKD are increasing in Asian populations [15]. It has also been suggested that the risk factor associations of CKD are different in Asians compared to whites. For example, in the KEEP-2 study in the US involving Asian Americans, Pacific islanders and whites, while hypertension was a significant risk factor for CKD in whites, Japanese, Native Hawaiians, Filipinos, no significant association was observed between hypertension and CKD in Chinese [16]. Given the ethnic differences in the prevalence of both hypertension and CKD [17] in Asian populations, it is important to clarify the association between BP and CKD in Asian ethnic groups. However, studies examining the association between BP and CKD in Asian populations have shown mixed results. While some studies involving Malays [18] and Japanese men [19] have reported an association between BP and CKD, some studies involving Chinese [20], Japanese [21] and Korean adults [22] have documented no significant association between BP and CKD. This inconsistency could partly be due to differences in methodology or population characteristics. To address this gap in literature, we aimed to examine the relationship between hypertension, individual BP components and CKD among Chinese, Malays and Indians in Singapore. The availability of three large datasets representing three largest Asian ethnic groups of similar age group, geographic region and study methods provides a unique opportunity to study the ethnic differences in the association between BP and CKD in Asia.

## Methods

### Study population

We evaluated data obtained from three large cross-sectional studies, Singapore Chinese Eye Study (SCES), Singapore Malay Eye Study (SiMES) and the Singapore Indian Eye Study (SINDI). SiMES, the first of the three studies was conducted between 2004–2006, followed by SINDI between 2007–2009 and the SCES between 2009–2011. All three studies followed the same study protocol and were conducted in the same study clinic (Singapore Eye Research Institute). All study participants were recruited from the southwestern part of Singapore. According to the 2000 Singapore census, the residents of southwestern part were a fair representation of the Singapore population in terms of age distribution and socioeconomic status. Details of the study population and methods of SiMES have been published elsewhere [23]. In brief, in SiMES, 5600 individuals were selected by an age-stratified random sampling method from the computer generated random list of 16,069 Malay names based on unique identity card number of the residents provided by the Ministry of Home Affairs. Of the 4,168 eligible individuals, 3280 participated in the study (78.7% response rate). Of the 3,148 participants with serum creatinine measurements, after excluding those with missing information on variables included in the multivariable model (n=66), 3,082 were included in the final analysis. In SINDI [24], 6,350 adults were selected by an age-stratified random sampling method from the computer generated random list of 11,616 Indian names provided by the Ministry of Home Affairs. Of the 4,497 eligible participants, 3,400 participated in the study (75.6% response rate). Of the 3,259 participants with serum creatinine measurements, after excluding those with missing information on variables included in the multivariable model (n=31), 3,228 provided data for the final analysis. In SCES [24], 6,752 adults were selected by an age-stratified random sampling method from the computer generated random list of 12,000 Chinese names provided by the Ministry of Home Affairs. Of the 4,605 eligible participants, 3,353 participated in the study (72.8% response rate). Of the 3,192 participants with serum creatinine measurements, after excluding those with missing information on variables included in the multivariable model (n=25), 3,167 provided data for the final analysis. Written informed consent was obtained from all participants and all studies were approved by the Singapore Eye Research Institute Institutional Review Board.

### Exposure measurement

Information on participants’ demographic characteristics, educational attainment, monthly income, cigarette smoking, alcohol consumption and medical history were obtained using a standardized questionnaire administered by trained personnel. BP measurement was taken with a digital automatic blood pressure monitor, (Dinamap model Pro Series DP110X-RW, 100V2; GE Medical Systems Information Technologies, Inc., USA) on 2 occasions 5 minutes apart, after the participants were seated for at least 5 minutes. If the blood pressures differed by more than 10 mm Hg systolic or 5 mm Hg diastolic, a third measurement was taken and BP of the individual was taken as the average of the two closest readings. Hypertension was defined as systolic BP ≥140 mm Hg or diastolic BP ≥90 mm Hg or self-reported previously diagnosed hypertension. BP was also categorized according to the JNC 7 categories [25] as 1) normal BP (<120 mm Hg systolic and <80 mm Hg diastolic); 2) prehypertension (120 to 139 mm Hg systolic or 80–89 mm Hg diastolic); 3) stage 1 hypertension (140 to 159 mm Hg systolic or 90 to 99 mm Hg diastolic); 4) stage 2 hypertension (≥160 mm Hg systolic or ≥100 mm Hg diastolic). All subjects on anti-hypertensive medication were classified as hypertensive irrespective of current BP. Thus those on antihypertensive medication but with ‘normal or prehypertensive range of BP’ were categorized under stage 1 hypertension and the rest were classified under stage 2 hypertension. Pulse pressure (PP) was defined as the difference between systolic BP and diastolic BP.

### Measurement of outcome

CKD was defined as an eGFR of <60 mL/min/1.73 m^2^ based on the US National Kidney Foundation Kidney Disease Outcome Quality Initiative (NKF-KDOQI) CKD stage 3 and above. GFR was estimated from serum creatinine using the recently developed Chronic Kidney Disease Epidemiology Collaboration (CKD-EPI) equation [26] as follows: 141 × min(S_cr_/k, 1)^α^× max(S_cr_/k, 1)^-1.209^×0.993^Age^ ×1.018 (for women), where S_cr_ is serum creatinine, k is 0.7 for females and 0.9 for males, α is −0.329 for females and −0.411 for males, min indicates the minimum of S_cr_/k or 1, and max indicates the maximum of S_cr_/k or 1. Serum creatinine was measured using an enzymatic method calibrated to the National Institute of Standards and Technology (NIST) Liquid Chromatography Isotope Dilution Mass Spectrometry (LC-IDMS) method recommended by the National Kidney Disease Education Program and traceable to NIST SRM967. Validation studies conducted in Singapore have shown CKD-EPI to be more accurate than MDRD in particular at higher eGFRs [27] and the prevalence of CKD by both MDRD and CKD-EPI to be similar in all three ethnic groups [28] suggesting the adoption of CKD-EPI equation without ethnic adjustment [27].

### Definition of other variables

Age was defined as the age at the time of examination and was categorized into 4 groups: 40–49, 50–59, 60–69, and 70–80. Education was used as an indicator of socioeconomic status and was categorized into 1) primary and below (≤6 years) 2) high school and above (>6 years). Income level was defined as individual gross income per month in Singapore dollars (SGD) and divided in to three categories: <1000, 1000 to <2000, ≥2000. Body mass index (BMI) was calculated as weight in kilograms divided by the square of height in meters (kg/m^2^). Diabetes mellitus was defined as a casual plasma glucose ≥200 mg/dl (11.1 mmol/L) or self-reported physician-diagnosed diabetes or use of glucose-lowering medication. Cigarette smoking was categorized into current, former and never smoker and alcohol consumption into drinkers and non-drinkers.

### Statistical analysis

We compared selected baseline characteristics of the study participants among the three ethnic groups using chi square test or analysis of variance, as appropriate for the variable. We examined the association of hypertension and JNC-7 categories of BP with CKD in three separate logistic regression models. In the first model we adjusted for age (years) and sex and in the multivariable model we additionally adjusted for categories of education, cigarette smoking, alcohol consumption, diabetes mellitus, and BMI (kg/m^2^). In the third model, we adjusted for antihypertensive medication use in addition to variables adjusted in the second model. We then examined the association of individual BP components (systolic, diastolic and PP) with CKD in the same regression models by categorizing BP in to quartiles of systolic BP, diastolic BP and PP. Since there were large differences in the distribution of systolic and diastolic BP among the three ethnic groups (p<0.0001), ethnicity specific quartiles were used for categorizing BP components. For this analysis, the lowest quartiles of systolic BP, diastolic BP and PP were used as the reference categories. Tests for trend were performed using the quartiles of BP as ordinal variables in corresponding regression models. We also analysed each component of BP as a continuous variable (per 10 units increase). Finally, we calculated the population attributable risk of CKD associated with hypertension in each ethnic group using Levin’s formula, Pe (RR-1)/(1+Pe (RR-1)) where Pe = Prevalence of exposure in the population [29,30]. We performed the analysis separately for each ethnic group. All statistical analyses were performed using SAS version 9.1.

## Results

Selected baseline characteristics of the three ethnic groups are shown in Table 1. Of the three ethnic groups, Chinese were older, less likely to be primary/below educated, and had lower levels of glucose, HbA1C and BMI levels and higher levels of eGFR than Malays and Indians. Malays were more likely to be primary & below educated, to have income < SGD 1000, and current smokers, had higher prevalence of hypertension, and overweight/obesity, had higher levels of systolic BP, diastolic BP and BMI and lower levels of eGFR and less likely to use antihypertensive medication; Indians were more likely to be ever drinkers, had higher prevalence of diabetes, higher levels of blood glucose, triglycerides and lower levels of total cholesterol and HDL cholesterol.

**Table 1:** Characteristics of the study participants by study cohort*

	**Chinese**	**Malay**	**Indian**	***P *value†**
	**(n=3,167)**	**(n=3,082)**	**(n=3,228)**	
Age, mean (SD), years	59.4 (9.8)	58.7 (11.0)	57.5 (10.0)	<0.0001
Gender, female, %	50.1	52.0	49.2	0.07
Primary & below education, %	52.5	75.3	55.6	<0.0001
Income <SGD 1000, %	46.1	68.8	48.2	<0.0001
Current smoking, %	13.0	20.2	14.9	<0.0001
Alcohol consumption, %	11.1	1.5	12.9	<0.0001
Hypertension, %	57.9	68.6	56.0	<0.0001
Antihypertensive medication use among those with previously diagnosed hypertension, %	89.8	53.4	83.1	<0.0001
Overweight/obesity, %	32.1	57.8	56.6	<0.0001
Blood glucose, mmol/L	6.4 (2.8)	6.8 (3.7)	7.2 (3.6)	<0.0001
Glycated hemoglobin (HbA1C), %	6.1 (0.9)	6.5 (1.6)	6.4 (1.4)	<0.0001
Systolic BP, mean (SD), mm Hg	136.2 (19.2)	147.2 (23.8)	135.2 (19.5)	<0.0001
Diastolic BP, mean (SD), mm Hg	77.6 (9.8)	79.8 (11.2)	77.5 (10.1)	<0.0001
Total cholesterol, mean (SD), mmol/L	5.5 (1.1)	5.6 (1.2)	5.2 (1.1)	<0.0001
HDL-cholesterol, mean (SD), mmol/L	1.3 (0.4)	1.4 (0.3)	1.1 (0.3)	<0.0001
Triglycerides, mean (SD), mmol/L	1.8 (1.3)	1.6 (1.3)	2.0 (1.2)	<0.0001
Body mass index, mean (SD), kg/m^2^	23.7 (3.6)	26.4 (5.1)	26.2 (4.8)	<0.0001
Estimated GFR, mL/min/1.73 m^2^	88.9 (18.5)	74.4 (20.4)	87.2 (18.1)	<0.0001

Abbreviations: HDL, high-density lipoprotein; GFR, glomerular filtration rate; SD, standard deviation.

* Data presented are row percentages or mean values and corresponding standard error.

†*P* value represents difference in characteristics by study cohort based on analysis of variance or chi square test as appropriate.

**Figure 1:**
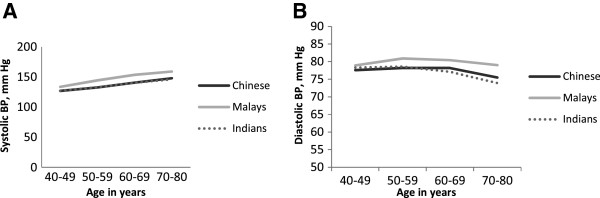
Distribution of systolic BP (1A) and diastolic BP (1B) by age groups across the three ethnic groups.

The distributions of SBP and DBP by age categories in the three ethnic groups are shown in Figure 1A and 1B. Systolic BP increased with age in all three populations; diastolic BP increased up to 60 years, plateau around 60 years and began to decline after 70 years in Chinese whereas diastolic BP increased up to 60 years and began to decline after 60 years in Malays and Indians. Systolic BP was consistently higher across all age groups in Malays as compared to Chinese and Indians. A similar higher trend for diastolic BP was also noted in Malays as compared to Chinese and Indians. The prevalence of CKD was highest among Malays (21.0%) followed by Indians (7.4%) and Chinese (5.9%). Table 2 shows the association between BP categories and CKD in the Chinese population. Majority of those with hypertension belonged to stage 1 hypertension. The prevalence of CKD increased with increasing JNC 7 BP categories and a significant graded association was observed across the JNC-7 BP categories in the multivariable model (p-trend =0.005). Among the individual JNC-7 categories, although none of the categories showed a significant statistical association, the direction of association with CKD was positive for stage 1 and 2 hypertension, but was negative for prehypertension. Presence of hypertension showed a significant positive association with CKD. Among the individual components, systolic BP and PP were not significantly associated with CKD in both quartile and continuous analysis. In contrast, diastolic BP was inversely associated with CKD in both models. However, this inverse association lost significance when we additionally adjusted for antihypertensive medication use.

**Table 2:** **Association between blood pressure (BP) categories and eGFR <60 mL/min/1.73 m**^**2 **^**in Chinese population**

**BP categories**	**No. at risk(cases)**	**Prevalence of eGFR <60 mL/min/1.73 m**^**2 **^**(%)**	**Age, sex-adjusted OR (95% CI)**	Multivariable model 1* OR (95% CI)	**Multivariable model 2† OR (95% CI)**
JNC-7 BP category‡					
Normal	492 (7)	1.4	1.00 (referent)	1.00 (referent)	-
Prehypertension	771 (10)	1.3	0.55 (0.20-1.50)	0.49 (0.18-1.35)	
Stage1 hypertension	1057 (88)	8.3	2.14 (0.95-4.82)	1.67 (0.73-3.81)	
Stage 2 hypertension	847 (115)	13.6	2.29 (1.01-5.18)	1.69 (0.73-3.90)	
P trend			0.002	0.005	
Hypertensive status					
Normotensive	1263 (17)	1.4	1.00 (referent)	1.00 (referent)	-
Hypertensive	1904 (203)	10.7	3.27 (1.94-5.53)	2.71 (1.59-4.63)	
Systolic BP quartiles					
Quartile 1 (82–122 mm Hg)	780 (32)	4.1	1.00 (referent)	1.00 (referent)	1.00 (referent)
Quartile 2 (123–135 mm Hg)	839 (46)	5.5	0.97 (0.59-1.61)	0.91 (0.54-1.52)	0.81 (0.48-1.37)
Quartile 3 (136–147 mm Hg)	754 (60)	8.0	0.89 (0.55-1.46)	0.82 (0.50-1.35)	0.75 (0.45-1.25)
Quartile 4 (148–225 mm Hg)	794 (82)	10.3	0.77 (0.48-1.25)	0.68 (0.42-1.11)	0.64 (0.39-1.07)
P trend			0.2	0.9	0.8
Each 10 units increase in systolic BP			0.96 (0.88-1.04)	0.94 (0.86-1.02)	0.94 (0.86-1.03)
Diastolic BP quartiles					
Quartile 1 (50–70 mm Hg)	869 (83)	9.6	1.00 (referent)	1.00 (referent)	1.00 (referent)
Quartile 2 (71–76 mm Hg)	723 (45)	6.2	0.53 (0.35-0.81)	0.55 (0.36-0.84)	0.53 (0.34-0.83)
Quartile 3 (77–83 mm Hg)	774 (54)	7.0	0.72 (0.48-1.07)	0.70 (0.47-1.04)	0.69 (0.44-1.08)
Quartile 4 (84–117 mm Hg)	801 (38)	4.7	0.53 (0.34-0.82)	0.53 (0.34-0.83)	0.63 (0.36-1.09)
P trend			0.01	0.01	0.1
Each 10 units increase in diastolic BP			0.82 (0.69-0.98)	0.81 (0.68-0.97)	0.87 (0.69-1.09)
Pulse pressure quartiles					
Quartile 1 (21–46 mm Hg)	786 (23)	2.9	1.00 (Referent)	1.00 (Referent)	1.00 (referent)
Quartile 2 (47–55 mm Hg)	775 (36)	4.7	0.96 (0.54-1.69)	0.90 (0.51-1.61)	0.85 (0.47-1.54)
Quartile 3 (56–67 mm Hg)	800 (59)	7.4	1.03 (0.60-1.77)	0.91 (0.53-1.58)	0.80 (0.45-1.39)
Quartile 4 (68–121 mm hg)	806 (102)	12.7	0.92 (0.53-1.58)	0.80 (0.46-1.39)	0.71 (0.40-1.24)
P trend			0.7	0.4	0.2
Each 10 units increase in pulse pressure			1.00 (0.90-1.10)	0.97 (0.88-1.08)	0.96 (0.87-1.07)

Abbreviations: eGFR, estimated glomerular filtration rate; OR, odds ratio; CI, confidence interval; * Adjusted for age (years), gender, education (primary and below, high school and above), body mass index (kg/m^2^), smoking (current, former, never), alcohol intake (present, absent), diabetes status (present, absent).

† Adjusted for variables in model 1 + antihypertensive medication use.

‡ BP classified according to Seventh Report of the Joint National Committee (JNC 7) on Prevention, Detection, Evaluation, and

Treatment of High Blood Pressure criteria (normal, systolic BP <120 mm Hg and diastolic BP <80 mm Hg; prehypertension,

systolic BP 120–139 or diastolic BP 80–89 mm Hg; stage 1 hypertension, systolic BP 140–159 or diastolic BP 90–99 mm Hg;

stage 2 hypertension, systolic BP ≥160 or diastolic BP ≥100 mm Hg).

Hypertension was defined as a self-reported current use of antihypertensive medication or systolic BP ≥140 mm Hg or diastolic BP ≥90 mm Hg.

Table 3 shows the association between BP categories and CKD in the Malay population. Majority of those with hypertension belonged to stage 2 hypertension. A significant graded association was observed across categories of JNC with CKD (p-trend <0.0001). Compared to those with normal BP, stage 1 and 2 hypertension showed a significant positive association with CKD. Prehypertension showed a positive association with CKD though short of significance. Presence of hypertension showed a significant positive association with CKD. All components of BP including systolic BP, diastolic BP and PP were significantly associated with CKD in both quartile and continuous analysis. This positive association between BP components and CKD remained unaltered with additional adjustment for antihypertensive medication.

**Table 3:** **Association between blood pressure (BP) categories and eGFR <60 mL/min/1.73 m**^**2 **^**in Malay population**

**BP categories**	**No. at risk (cases)**	**Prevalence of eGFR <60 mL/min/1.73 m**^**2 **^**(%)**	**Age, sex-adjusted OR (95% CI)**	Multivariable model 1* OR (95% CI)	**Multivariable model 2† OR (95% CI)**
JNC-7 BP category‡					
Normal	300 (15)	5.0	1.00 (referent)	1.00 (referent)	-
Prehypertension	757 (84)	11.1	1.65 (0.92-2.97)	1.55 (0.85-2.81)	
Stage1 hypertension	877 (181)	20.6	2.51 (1.43-4.43)	2.25 (1.27-4.01)	
Stage 2 hypertension	1148 (406)	35.4	4.19 (2.40-7.32)	3.58 (2.03-6.32)	
P trend			<0.0001	<0.0001	
Hypertensive status					
Normotensive	1057 (99)	9.4	1.00 (referent)	1.00 (referent)	-
Hypertensive	2025 (587)	29.0	2.26 (1.77-2.90)	2.08 (1.62-2.68)	
Systolic BP quartiles					
Quartile 1 (84–129 mm Hg)	761 (81)	10.6	1.00 (referent)	1.00 (referent)	1.00 (referent)
Quartile 2 (130–143 mm Hg)	744 (132)	17.7	1.40 (1.02-1.92)	1.29 (0.94-1.79)	1.25 (0.90-1.73)
Quartile 3 (144–162 mm Hg)	7987 (196)	24.6	1.71 (1.26-2.31)	1.53 (1.12-2.09)	1.46 (1.07-2.00)
Quartile 4 (163–275 mm Hg)	7794 (277)	35.6	2.19 (1.63-2.95)	1.97 (1.45-2.66)	1.94 (1.43-2.64)
P trend			<0.0001	<0.0001	<0.0001
Each 10 units increase in systolic BP			1.13 (1.09-1.18)	1.11 (1.07-1.16)	1.12 (1.07-1.17)
Diastolic BP quartiles					
Quartile 1 (48–70 mm Hg)	727 (152)	20.9	1.00 (referent)	1.00 (referent)	1.00 (referent)
Quartile 2 (71–77 mm Hg)	752 (172)	22.9	1.04 (0.79-1.36)	1.05 (0.80-1.38)	1.06 (0.80-1.39)
Quartile 3 (78–86 mm Hg)	833 (173)	20.8	1.02 (0.78-1.33)	1.00 (0.76-1.32)	1.03 (0.78-1.36)
Quartile 4 (87–131 mm Hg)	770 (189)	24.6	1.27 (0.97-1.66)	1.31 (0.99-1.72)	1.39 (1.05-1.83)
P trend			0.1	0.08	0.03
Each 10 units increase in diastolic BP			1.09 (1.00-1.19)	1.10 (1.01-1.20)	1.13 (1.04-1.23)
Pulse pressure quartiles					
Quartile 1 (26–52 mm Hg)	758 (64)	8.4	1.00 (referent)	1.00 (referent)	1.00 (referent)
Quartile 2 (53–65 mm Hg)	781 (130)	16.7	1.47 (1.05-2.07)	1.33 (0.94-1.88)	1.30 (0.91-1.84)
Quartile 3 (66–79 mm Hg)	765 (192)	25.1	1.82 (1.31-2.54)	1.51 (1.07-2.12)	1.45 (1.03-2.04)
Quartile 4 (80–136 mm hg)	778 (300)	38.6	2.58 (1.85-3.61)	2.10 (1.49-2.95)	2.04 (1.44-2.87)
P trend			<0.0001	<0.0001	<0.0001
Each 10 units increase in pulse pressure			1.21 (1.14-1.28)	1.17 (1.10-1.24)	1.16 (1.09-1.23)

Abbreviations: eGFR, estimated glomerular filtration rate; OR, odds ratio; CI, confidence interval; * Adjusted for age (years), gender, education (primary and below, high school and above), body mass index (kg/m^2^), smoking (current, former, never), alcohol intake (present, absent), diabetes status (present, absent).

† Adjusted for variables in model 1 + antihypertensive medication use.

‡ BP classified according to Seventh Report of the Joint National Committee (JNC 7) on Prevention, Detection, Evaluation, and

Treatment of High Blood Pressure criteria (normal, systolic BP <120 mm Hg and diastolic BP <80 mm Hg; prehypertension,

systolic BP 120–139 or diastolic BP 80–89 mm Hg; stage 1 hypertension, systolic BP 140–159 or diastolic BP 90–99 mm Hg;

stage 2 hypertension, systolic BP ≥160 or diastolic BP ≥100 mm Hg).

Hypertension was defined as a self-reported current use of antihypertensive medication or systolic BP ≥140 mm Hg or diastolic BP ≥90 mm Hg.

Table 4 shows the association between BP categories and CKD in the Indian population. Majority of those with hypertension had stage 1 hypertension. A significant graded association was observed between JNC-7 categories and CKD (p-trend = 0.001) and stage 1 and 2 hypertension showed a significant positive association with CKD. Similar to Chinese, prehypertension showed a non-significant negative association with CKD. Presence of hypertension showed a significant positive association with CKD similar to the other two ethnic groups. Among the individual components, systolic BP was not significantly associated with CKD in both quartile and continuous analysis. In contrast, diastolic BP was inversely associated with CKD in both age, sex-adjusted and the first multivariable model. However, this inverse association lost significance with additional adjustment to antihypertensive medication use. Similarly, pulse pressure showed a positive association with CKD in quartile and continuous analysis in the age, sex-adjusted and the first multivariable model. But the association became insignificant with additional adjustment for antihypertensive medication. In Table 5, we have summarized the effect estimates of CKD associated with hypertension and BP components in the three ethnic groups. In PAR calculations, the proportion of CKD prevalence that can be attributable to hypertension was 49.8% among Chinese, 42.6% among Malays and 44.4% among Indians.

**Table 4:** **Association between blood pressure (BP) categories and eGFR <60 mL/min/1.73 m**^**2 **^**in Indian population**

**BP categories**	**No. at risk (cases)**	**Prevalence of eGFR <60 mL/min/1.73 m**^**2 **^**(%)**	**Age, sex-adjusted OR (95% CI)**	Multivariable model 1* OR (95% CI)	**Multivariable model 2† OR (95% CI)**
JNC-7 BP category‡					
Normal	532 (12)	2.3	1.00 (referent)	1.00 (referent)	-
Prehypertension	834 (24)	2.9	0.98 (0.48-2.00)	0.87 (0.42-1.78)	
Stage1 hypertension	1052 (122)	11.6	2.98 (1.60-5.54)	2.40 (1.28-4.51)	
Stage 2 hypertension	810 (106)	13.1	2.63 (1.40-4.95)	1.98 (1.04-3.78)	
P trend		8.2	<0.0001	0.002	
Hypertensive status					
Normotensive	1366 (36)	2.6	1.00 (referent)	1.00 (referent)	-
Hypertensive	1862 (228)	12.2	2.86 (1.97-4.16)	2.43 (1.66-3.57)	
Systolic BP quartiles					
Quartile 1 (79–121 mm Hg)	825 (42)	5.1	1.00 (referent)	1.00 (referent)	1.00 (referent)
Quartile 2 (122–133 mm Hg)	782 (64)	8.2	1.19 (0.78-1.82)	1.11 (0.73-1.71)	1.06 (0.69-1.64)
Quartile 3 (134–147 mm Hg)	817 (54)	6.6	0.76 (0.49-1.18)	0.68 (0.44-1.07)	0.61 (0.39-0.96)
Quartile 4 (148–237 mm Hg)	804 (104)	13.0	1.24 (0.83-1.84)	1.06 (0.71-1.59)	0.96 (0.64-1.45)
P trend			0.5	0.9	0.5
Each 10 units increase in systolic BP			1.00 (0.94-1.08)	0.98 (0.91-1.05)	0.97 (0.90-1.04)
Diastolic BP quartiles					
Quartile 1 (43–69 mm Hg)	810 (102)	12.6	1.00 (referent)	1.00 (referent)	1.00 (referent)
Quartile 2 (70–76 mm Hg)	803 (72)	9.0	0.69 (0.49-0.96)	0.74 (0.51-1.06)	0.81 (0.56-1.16)
Quartile 3 (77–83 mm Hg)	786 (52)	6.6	0.55 (0.38-0.79)	0.64 (0.42-0.97)	0.78 (0.52-1.17)
Quartile 4 (84–136 mm Hg)	829 (38)	4.6	0.42 (0.28-0.63)	0.54 (0.32-0.90)	0.71 (0.45-1.13)
P trend			<0.0001	0.01	0.1
Each 10 units increase in diastolic BP			0.67 (0.58-0.79)	0.71 (0.58-0.88)	0.83 (0.70-1.00)
Pulse pressure quartiles					
Quartile 1 (23–45 mm Hg)	804 (23)	2.9	1.00 (Referent)	1.00 (Referent)	1.00 (referent)
Quartile 2 (46–55 mm Hg)	768 (45)	5.9	1.53 (0.89-2.57)	1.42 (0.83-2.42)	1.40 (0.82-2.41)
Quartile 3 (56–67 mm Hg)	846 (72)	8.5	1.62 (0.98-2.68)	1.35 (0.81-2.25)	1.22 (0.72-2.04)
Quartile 4 (68–121 mm hg)	810 (124)	15.3	2.15 (1.31-3.52)	1.66 (1.00-2.76)	1.46 (0.87-2.43)
P trend			0.002	0.07	0.2
Each 10 units increase in pulse pressure			1.15 (1.06-1.25)	1.09 (1.00-1.19)	1.06 (0.97-1.16)

Abbreviations: eGFR, estimated glomerular filtration rate; OR, odds ratio; CI, confidence interval.

* Adjusted for age (years), gender, education (primary and below, high school and above), body mass index (kg/m^2^), smoking (current, former, never), alcohol intake (present, absent), diabetes status (present, absent).

† Adjusted for variables in model 1 + antihypertensive medication use.

‡ BP classified according to Seventh Report of the Joint National Committee (JNC 7) on Prevention, Detection, Evaluation, and

Treatment of High Blood Pressure criteria (normal, systolic BP <120 mm Hg and diastolic BP <80 mm Hg; prehypertension,

systolic BP 120–139 or diastolic BP 80–89 mm Hg; stage 1 hypertension, systolic BP 140–159 or diastolic BP 90–99 mm Hg;

stage 2 hypertension, systolic BP ≥160 or diastolic BP ≥100 mm Hg).

Hypertension was defined as a self-reported current use of antihypertensive medication or systolic BP ≥140 mm Hg or diastolic BP ≥90 mm Hg.

**Table 5:** Summary of odds ratio of CKD for BP components by ethnicity

**Ethnic groups**	Multivariable model 1 OR (95% CI)*	**Multivariable model 2 OR (95% CI) †**
Hypertension (normotensive Vs. hypertensive)		
Chinese	3.27 (1.94-5.53)	2.71 (1.59-4.63)
Malays	2.26 (1.77-2.90)	2.08 (1.62-2.68)
Indians	2.86 (1.97-4.16)	2.43 (1.66-3.57)
Systolic BP (Q4 Vs. Q1)		
Chinese	0.68 (0.42-1.11)	0.64 (0.39-1.07)
Malays	1.97 (1.45-2.66)	1.94 (1.43-2.64)
Indians	1.06 (0.71-1.59)	0.96 (0.64-1.45)
Diastolic BP (Q4 Vs. Q1)		
Chinese	0.53 (0.34-0.83)	0.63 (0.36-1.09)
Malays	1.31 (0.99-1.72)	1.39 (1.05-1.83)
Indians	0.54 (0.32-0.90)	0.71 (0.45-1.13)

Abbreviations: BP, blood pressure; CI, confidence interval; OR, odds ratio; Q, quartile.

* Adjusted for age, gender, education, body mass index, smoking, alcohol intake, diabetes.

† Adjusted for variables in model 1 + antihypertensive medication use.

## Discussion

In this study, examining the association between BP and CKD using data from three independent studies of Chinese, Malay and Indian ethnic groups aged 40–80 years in Singapore, we found that although hypertension was associated with CKD in all three ethnic groups, the pattern of association between individual BP components and CKD varied across ethnic groups. We found that in Malays, all BP components including systolic BP, diastolic BP and PP were significantly associated with CKD. In Chinese and Indians, systolic BP and PP did not show a significant association whereas diastolic BP showed an inverse association with CKD which was explained by anti-hypertensive medication use in both ethnic groups.

We found that the prevalence of both hypertension and CKD were higher among Malays compared to Chinese and Indians consistent with a recent report from the Singapore National Health Survey (NHS) 2010 [31], and from a cross-sectional study in Malaysia [32]. In addition, the pattern of distribution of risk factors of CKD across the three ethnic groups observed in the current study closely followed that reported by the recent NHS [31]. For example, the prevalence of current smoking and obesity was higher among Malays and the prevalence of diabetes was higher and levels of HDL cholesterol were lower among Indians. These consistent findings with previous reports suggest that our results have sufficient internal validity.

In the current study, presence of hypertension was associated with CKD in all three ethnic groups with the PAR of hypertension being 49.8%, 42.6% and 44.4% in Chinese, Malays and Indians. Hypertension has consistently been shown to be an important risk factor for CKD in Western populations [7,8,13,33]. While majority of the studies involving Asian populations [19,34-36] have reported an association between hypertension and CKD, few studies involving Asian Chinese in the US [16], a large cohort of adults in Japan [21] and Korea [22] have documented no significant association of hypertension with CKD. Although hypertension has been shown to be an important risk factor for ESRD in the US general population, ethnic differences have been reported in the association between hypertension and ESRD with blacks having a 4–5 fold increased risk of hypertensive ESRD compared to whites [37,38]. However, in the current study, the magnitude of association between hypertension and CKD was similar (OR ranging from 2.08 to 2.71) across the three ethnic groups. In addition to hypertension categories, studies conducted in the Western populations reported a positive association between prehypertension and CKD [8,39-41]. Consistent with previous reports, in the current study, a weak positive association was found between prehypertension and CKD only in Malays.

In the current study, systolic BP was associated with CKD only in Malays. Of all the BP components, systolic BP has been shown to be the most useful predictor of CKD in both general [3,10,12,13] as well as high-risk populations [42]. Further, systolic BP predicts progression of CKD [9,43,44] and adverse outcomes [45,46] among those with pre-existing CKD. Systolic BP was shown to be a significant predictor of CKD among male Physicians in the Physicians Health Study (PHS) [13], in a large cohort of men in the MRFIT study [10] and in an elderly cohort with isolated systolic hypertension in the US [12]. Two reviews of observational epidemiological studies and clinical trials reported a graded association between systolic BP and CKD and a reduced risk of CKD with systolic BP reduction [3,46]. In the Kidney Disease Early Evaluation Program (KEEP) involving African Americans at-risk for CKD, systolic BP was associated with both early and late stages of CKD [42]. Systolic BP has also been shown to be associated with progression of CKD in a small cohort of male veterans [9], a large cohort of KEEP participants [11], and in a large cohort of adults in the REasons for the Geographic And Racial Differences in Stroke study [43] with preexisting CKD. While studies involving Western populations have shown a consistent positive association between systolic BP and CKD, the pattern of association of systolic BP with CKD has not been consistent in Asian populations. Systolic BP was shown to be strong predictor of ESRD in a large cohort of Chinese adults who participated in the China National Hypertension Survey Epidemiology Follow-up Study (CHEFS) [44] and in a large cohort of middle-aged Japanese men who participated in the Kansai Healthcare Study in Japan [19]. However, similar to our findings in Chinese and Indians, three cross-sectional studies involving Chinese [20], and Japanese [21,47] adults reported no significant association between systolic BP and CKD.

We found that elevated levels of diastolic BP were associated with CKD in Malays only. Compared to systolic BP, diastolic BP has been shown to be less predictive of CKD and adverse outcomes in CKD in majority of the studies conducted in Western populations [9,13,43,48]. In the PHS, of all the BP components, diastolic BP was found to be less predictive of CKD [13]. In the Reduction in endpoints in NIDDM with the Angiotensin II Antagonist Losartan (RENAAL) Study, an increased risk for ESRD was observed for systolic BP only [48]. Similar results were observed in a prospective study of veterans with CKD by Agarwal et al. in the US [9]. In the REGARDS, for a 11 mm increased in diastolic BP, the risk of ESRD increased by 38% [43], however, when systolic and diastolic BP were considered together, the association of diastolic BP with ESRD lost significance [43]. In the KEEP study, diastolic BP greater than 90 mm Hg was associated with an increased risk for ESRD in subjects with CKD [11]. Similar to our findings in Malays, majority of the studies involving Asian populations have shown an association between diastolic BP and CKD [19,21,49] and ESRD [44,50]. Diastolic BP was found to be associated with CKD in a large cohort of Koreans [49], and two cohorts of Japanese [19,21]. Similar association between diastolic BP and ESRD was observed in a large cohort of Japanese [50] Chinese adults [44]. In the current study, elevated diastolic BP showed an inverse association with CKD, in other words, low diastolic BP was associated with CKD in Chinese and Indians. Although this protective association lost its statistical significance after additional adjustment for antihypertensive medication use in Chinese, and Indians, the direction of association still remained the same. The reason for the protective association of diastolic BP with CKD Chinese, and Indians is not clear. Recent studies have shown that among those with higher systolic BP, lower diastolic BP, otherwise wider pulse pressure indicating large arterial stiffening increases the risk of CVD [51,52]. Accordingly, when we explored the relationship between systolic and diastolic BP among the study participants, we found that 76% of Chinese, 86% of Malays and 71% of Indians had systolic BP ≥130 mm Hg; 21% of Chinese and Indians and 29% of Malays had diastolic BP ≥85 mm Hg; in all three ethnic groups, over 90% of those with higher diastolic BP had higher systolic BP. Hence it is conceivable that higher diastolic BP present among majority of participants with higher systolic BP is protective against CKD. Tozawa et al. has shown that use of antihypertensive medication correlated positively with wider PP in hemodialysis patients [36]. Peralta et al. have shown that treatment of hypertension with multiple anti-hypertensive drugs is associated with lowering of diastolic BP more than systolic BP among CKD subjects [53]. Low diastolic BP in dialysis patients has been shown to be associated with poorer outcomes [54]. In the Framingham Heart Study, lower diastolic BP at any level of systolic BP above 120 mm Hg increased the risk of coronary heart disease in middle aged and elderly adults [55]. In a small cohort of male veterans with CKD in the US, Agarwal et al. has shown an inverse association between diastolic BP and all-cause mortality [9].

In the current study, PP was associated with CKD in Malays only. The initial association of PP with CKD lost significance after adjusting for anti-hypertensive medication use in Indians. PP, a measure of arterial stiffness has been shown to be associated with CKD [13], and progression of CKD including ESRD in several studies [11,43,48,56]. Higher PP and low diastolic BP has been shown to be associated adverse cardiovascular outcomes in the Framingham Heart Study [55].

In the current study, Malays had higher levels of systolic and diastolic BP at all age groups and higher prevalence of CKD than Chinese and Indians. While hypertension was positively associated with CKD in all three ethnic groups, BP components including systolic BP, diastolic BP and PP were positively associated with CKD only in Malays. In Chinese and Indians, while systolic BP and PP did not show a significant association, diastolic BP showed an inverse association with CKD that could possibly be explained by the higher proportion of antihypertensive medication use in Chinese and Indians. It is possible that a higher prevalence of underlying risk factors for both hypertension and CKD may have influenced the positive association of BP with CKD in Malays. Although Malays and Indians both had similar adverse metabolic risk profile (lipids, diabetes and obesity) compared to Chinese, Indians had lower prevalence of CKD. This is consistent with a previous study in Singapore that reported Malay ethnicity to be associated with a higher prevalence of proteinuria [57] and also consistent with reports from India that showed a lower prevalence of diabetic retinopathy [58] and nephropathy [59] among Indians suggesting that Indians may be less susceptible to microvascular complications [58,59]. In addition, socioeconomic differentials across the three ethnic groups contributing to poor BP control despite treatment [60], and obesity [61] may have also influenced the association of BP with CKD.

Our study has some limitations. First, because of the cross-sectional analysis, the temporal association between BP and CKD could not be elucidated and reverse causality is possible. Second, we did not examine the effect of the type of antihypertensive medication in the association between BP and CKD as the information on the type of medication was incomplete. Third, our assessment of BP on a single occasion could have caused a non-differential misclassification of BP status. Fourth, we did not have information on microalbuminuria, another indicator of CKD, which is also associated with diabetes and hypertension. Therefore, we were not able to study the impact of BP on microalbuminuria. Finally, while response rates of those invited to participate in the three studies were excellent (73%-79%), not all subjects had the necessary data available for the current analysis. Given the differences in baseline characteristics such as education level between ethnic groups, it is possible for non-participation or missing data to introduce a selection bias. However, the consistency of our findings on risk factor profile of the three ethnic groups with previously published reports suggests that this bias is less likely to influence our findings. The large sample size and the availability of three independent studies representing three major Asian ethnic groups with similar methodology and objective assessment of exposure and outcome measurements are the strengths of our study.

## Conclusion

In conclusion, in a large sample of Asian adults representing three major ethnic groups, we found that the pattern of association of BP components with CKD was different across the three ethnic groups. All three components of BP including systolic BP, diastolic BP and PP were associated with CKD in Malays. In Chinese and Indians, systolic BP was not associated with CKD while diastolic BP showed an inverse association that was explained by antihypertensive medication use. Our findings in Chinese and Indians may have implications for anti-hypertensive treatment in CKD patients if confirmed by future prospective studies.

## Competing interests

The authors declare that they have no competing interests.

## Authors’ contributions

All authors contributed to the intellectual development of this paper. CS, BWT, EST had the original idea for the study. CS analyzed the data, wrote the first draft paper. BWT, EST, THJ, TYW provided statistical expertise and critical corrections to the manuscript. TYW supervised data collection. All authors read and approved the final manuscript.

## Pre-publication history

The pre-publication history for this paper can be accessed here:

http://www.biomedcentral.com/1471-2369/14/86/prepub

## References

[B1] Whelton PK Klag MJ Hypertension as a risk factor for renal disease. Review of clinical and epidemiological evidence Hypertension 1989 13 I19 I27 10.1161/01.HYP.13.5_Suppl.I19 2490824

[B2] Barri YM Hypertension and kidney disease: a deadly connection Curr Cardiol Rep 2006 8 411 417 10.1007/s11886-006-0098-7 17059792

[B3] Whelton PK Perneger TV He J Klag MJ The role of blood pressure as a risk factor for renal disease: a review of the epidemiologic evidence J Hum Hypertens 1996 10 683 689 9004095

[B4] Jafar TH Stark PC Schmid CH Landa M Maschio G de Jong PE Progression of chronic kidney disease: the role of blood pressure control, proteinuria, and angiotensin-converting enzyme inhibition: a patient-level meta-analysis Ann Intern Med 2003 139 244 252 10.7326/0003-4819-139-4-200308190-00006 12965979

[B5] Peterson JC Adler S Burkart JM Greene T Hebert LA Hunsicker LG Blood pressure control, proteinuria, and the progression of renal disease. The Modification of Diet in Renal Disease Study Ann Intern Med 1995 123 754 762 10.7326/0003-4819-123-10-199511150-00003 7574193

[B6] Coresh J Selvin E Stevens LA Manzi J Kusek JW Eggers P Prevalence of chronic kidney disease in the United States JAMA 2007 298 2038 2047 10.1001/jama.298.17.2038 17986697

[B7] Haroun MK Jaar BG Hoffman SC Comstock GW Klag MJ Coresh J Risk factors for chronic kidney disease: a prospective study of 23,534 men and women in Washington County, Maryland J Am Soc Nephrol 2003 14 2934 2941 10.1097/01.ASN.0000095249.99803.85 14569104

[B8] Hsu CY McCulloch CE Darbinian J Go AS Iribarren C Elevated blood pressure and risk of end-stage renal disease in subjects without baseline kidney disease Arch Intern Med 2005 165 923 928 10.1001/archinte.165.8.923 15851645

[B9] Agarwal R Blood pressure components and the risk for end-stage renal disease and death in chronic kidney disease Clin J Am Soc Nephrol 2009 4 830 837 10.2215/CJN.06201208 19339424 PMC2666439

[B10] Klag MJ Whelton PK Randall BL Neaton JD Brancati FL Ford CE Blood pressure and end-stage renal disease in men N Engl J Med 1996 334 13 18 10.1056/NEJM199601043340103 7494564

[B11] Peralta CA Norris KC Li S Chang TI Tamura MK Jolly SE Blood pressure components and end-stage renal disease in persons with chronic kidney disease: the Kidney Early Evaluation Program (KEEP) Arch Intern Med 2012 172 41 47 10.1001/archinternmed.2011.619 22232147 PMC3417125

[B12] Young JH Klag MJ Muntner P Whyte JL Pahor M Coresh J Blood pressure and decline in kidney function: findings from the Systolic Hypertension in the Elderly Program (SHEP) J Am Soc Nephrol 2002 13 2776 2782 10.1097/01.ASN.0000031805.09178.37 12397049

[B13] Schaeffner ES Kurth T Bowman TS Gelber RP Gaziano JM Blood pressure measures and risk of chronic kidney disease in men Nephrol Dial Transplant 2008 23 1246 1251 17984108 10.1093/ndt/gfm757

[B14] Hall YN Hsu CY Iribarren C Darbinian J McCulloch CE Go AS The conundrum of increased burden of end-stage renal disease in Asians Kidney Int 2005 68 2310 2316 10.1111/j.1523-1755.2005.00691.x 16221234

[B15] Jafar TH Hypertension and kidney disease in Asia Curr Opin Nephrol Hypertens 2006 15 291 295 10.1097/01.mnh.0000222697.30207.4e 16609297

[B16] Mau MK West MR Shara NM Efird JT Alimineti K Saito E Epidemiologic and clinical factors associated with chronic kidney disease among Asian Americans and Native Hawaiians Ethn Health 2007 12 111 127 10.1080/13557850601081720 17364897

[B17] Sabanayagam C Lim SC Wong TY Lee J Shankar A Tai ES Ethnic disparities in prevalence and impact of risk factors of chronic kidney disease Nephrol Dial Transplant 2010 25 2564 2570 10.1093/ndt/gfq084 20185856

[B18] Sabanayagam C Shankar A Lim SC Tai ES Wong TY Hypertension, hypertension control, and chronic kidney disease in a Malay population in Singapore Asia Pac J Public Health 2011 23 936 945 10.1177/1010539510361637 20460283

[B19] Koh H Hayashi T Sato KK Harita N Maeda I Nakamura Y Blood pressure components and risk for chronic kidney disease in middle-aged Japanese men: The Kansai Healthcare Study Hypertens Res 2011 34 536 541 10.1038/hr.2011.2 21270813

[B20] Chen J Gu D Chen CS Wu X Hamm LL Muntner P Association between the metabolic syndrome and chronic kidney disease in Chinese adults Nephrol Dial Transplant 2007 22 1100 1106 10.1093/ndt/gfl759 17272313

[B21] Kawamoto R Kohara K Tabara Y Miki T An association between metabolic syndrome and the estimated glomerular filtration rate Intern Med 2008 47 1399 1406 10.2169/internalmedicine.47.1202 18670145

[B22] Jang SY Kim IH Ju EY Ahn SJ Kim DK Lee SW Chronic kidney disease and metabolic syndrome in a general Korean population: the Third Korea National Health and Nutrition Examination Survey (KNHANES III) Study J Public Health (Oxf) 2010 32 538 546 10.1093/pubmed/fdp127 20061374

[B23] Foong AW Saw SM Loo JL Shen S Loon SC Rosman M Rationale and methodology for a population-based study of eye diseases in Malay people: The Singapore Malay eye study (SiMES) Ophthalmic Epidemiol 2007 14 25 35 10.1080/09286580600878844 17365815

[B24] Lavanya R Jeganathan VS Zheng Y Raju P Cheung N Tai ES Methodology of the Singapore Indian Chinese Cohort (SICC) eye study: quantifying ethnic variations in the epidemiology of eye diseases in Asians Ophthalmic Epidemiol 2009 16 325 336 10.3109/09286580903144738 19995197

[B25] Chobanian AV Bakris GL Black HR Cushman WC Green LA Izzo JL Jr The Seventh Report of the Joint National Committee on Prevention, Detection, Evaluation, and Treatment of High Blood Pressure: the JNC 7 report JAMA 2003 289 2560 2572 10.1001/jama.289.19.2560 12748199

[B26] Levey AS Stevens LA Schmid CH Zhang YL Castro AF III Feldman HI A new equation to estimate glomerular filtration rate Ann Intern Med 2009 150 604 612 10.7326/0003-4819-150-9-200905050-00006 19414839 PMC2763564

[B27] Teo BW Xu H Koh YY Li J Sinha AK Shuter B Estimating kidney function in a multiethnic Asian population with multiple filtration markers Am J Kidney Dis 2012 60 500 502 10.1053/j.ajkd.2012.05.008 22721932

[B28] Sabanayagam C Wong TY Tai ES The CKD-EPI equation and MDRD study equation find similar prevalence of chronic kidney disease in Asian populations Ann Intern Med 2009 151 892 893 20008766 10.7326/0003-4819-151-12-200912150-00014

[B29] Walter SD The estimation and interpretation of attributable risk in health research Biometrics 1976 32 829 849 10.2307/2529268 1009228

[B30] Moss ME Lanphear BP Auinger P Association of dental caries and blood lead levels JAMA 1999 281 2294 2298 10.1001/jama.281.24.2294 10386553

[B31] Ministry of Health S National Health Survey 2010 [article online]. http://www.moh.gov.sg/content/dam/moh_web/Publications/Reports/2011/NHS2010%20-%20low%20respdf [accessed Oct 28, 2012] 2011, 1–13

[B32] Rampal L Rampal S Azhar MZ Rahman AR Prevalence, awareness, treatment and control of hypertension in Malaysia: a national study of 16,440 subjects Public Health 2008 122 11 18 10.1016/j.puhe.2007.05.008 17981310

[B33] Coresh J Wei GL McQuillan G Brancati FL Levey AS Jones C Prevalence of high blood pressure and elevated serum creatinine level in the United States: findings from the third National Health and Nutrition Examination Survey (1988–1994) Arch Intern Med 2001 161 1207 1216 10.1001/archinte.161.9.1207 11343443

[B34] Yamagata K Ishida K Sairenchi T Takahashi H Ohba S Shiigai T Risk factors for chronic kidney disease in a community-based population: a 10-year follow-up study Kidney Int 2007 71 159 166 10.1038/sj.ki.5002017 17136030

[B35] Sabanayagam C Tai ES Shankar A Lee J Sun C Wong TY Retinal arteriolar narrowing increases the likelihood of chronic kidney disease in hypertension J Hypertens 2009 27 2209 2217 10.1097/HJH.0b013e328330141d 19620884

[B36] Tozawa M Iseki K Iseki C Kinjo K Ikemiya Y Takishita S Blood pressure predicts risk of developing end-stage renal disease in men and women Hypertension 2003 41 1341 1345 10.1161/01.HYP.0000069699.92349.8C 12707291

[B37] Whittle JC Whelton PK Seidler AJ Klag MJ Does racial variation in risk factors explain black-white differences in the incidence of hypertensive end-stage renal disease? Arch Intern Med 1991 151 1359 1364 10.1001/archinte.1991.00400070121015 2064486

[B38] Klag MJ Whelton PK Randall BL Neaton JD Brancati FL Stamler J End-stage renal disease in African-American and white men. 16-year MRFIT findings JAMA 1997 277 1293 1298 10.1001/jama.1997.03540400043029 9109467

[B39] Perneger TV Nieto FJ Whelton PK Klag MJ Comstock GW Szklo M A prospective study of blood pressure and serum creatinine. Results from the ‘Clue’ Study and the ARIC Study JAMA 1993 269 488 493 10.1001/jama.1993.03500040054036 8419668

[B40] Crews DC Plantinga LC Miller ER III Saran R Hedgeman E Saydah SH Prevalence of chronic kidney disease in persons with undiagnosed or prehypertension in the United States Hypertension 2010 55 1102 1109 10.1161/HYPERTENSIONAHA.110.150722 20308607 PMC2880533

[B41] Yan P Zhu X Li H Shrubsole MJ Shi H Zhang MZ Association of high blood pressure with renal insufficiency: role of albuminuria, from NHANES, 1999–2006 PLoS One 2012 7 e37837 10.1371/journal.pone.0037837 22802927 PMC3388992

[B42] Kalaitzidis R Li S Wang C Chen SC McCullough PA Bakris GL Hypertension in early-stage kidney disease: an update from the Kidney Early Evaluation Program (KEEP) Am J Kidney Dis 2009 53 S22 S31 10.1053/j.ajkd.2008.11.028 19285608

[B43] Bell EK Gao L Judd S Glasser SP McClellan W Gutierrez OM Blood pressure indexes and end-stage renal disease risk in adults with chronic kidney disease Am J Hypertens 2012 25 789 796 10.1038/ajh.2012.48 22573012 PMC3784349

[B44] Reynolds K Gu D Muntner P Kusek JW Chen J Wu X A population-based, prospective study of blood pressure and risk for end-stage renal disease in china J Am Soc Nephrol 2007 18 1928 1935 10.1681/ASN.2006111199 17475822

[B45] Cutler JA High blood pressure and end-organ damage J Hypertens Suppl 1996 14 S3 S6 9023707

[B46] He J Whelton PK Elevated systolic blood pressure and risk of cardiovascular and renal disease: overview of evidence from observational epidemiologic studies and randomized controlled trials Am Heart J 1999 138 211 219 10.1016/S0002-8703(99)70312-1 10467215

[B47] Higashikuni Y Ishizaka N Ishizaka Y Toda E Nagai R Yamakado M Relationship between blood pressure and chronic kidney disease in the Japanese population: the lower the better even in individuals without hypertension? Hypertens Res 2008 31 213 219 10.1291/hypres.31.213 18360039

[B48] Bakris GL Weir MR Shanifar S Zhang Z Douglas J van Dijk DJ Effects of blood pressure level on progression of diabetic nephropathy: results from the RENAAL study Arch Intern Med 2003 163 1555 1565 10.1001/archinte.163.13.1555 12860578

[B49] Kim S Lim CS Han DC Kim GS Chin HJ Kim SJ The prevalence of chronic kidney disease (CKD) and the associated factors to CKD in urban Korea: a population-based cross-sectional epidemiologic study J Korean Med Sci 2009 24 Suppl S11 S21 10.3346/jkms.2009.24.S1.S11 19194539 PMC2633200

[B50] Iseki K Iseki C Ikemiya Y Fukiyama K Risk of developing end-stage renal disease in a cohort of mass screening Kidney Int 1996 49 800 805 10.1038/ki.1996.111 8648923

[B51] Benetos A Zureik M Morcet J Thomas F Bean K Safar M A decrease in diastolic blood pressure combined with an increase in systolic blood pressure is associated with a higher cardiovascular mortality in men J Am Coll Cardiol 2000 35 673 680 10.1016/S0735-1097(99)00586-0 10716470

[B52] Benetos A Thomas F Safar ME Bean KE Guize L Should diastolic and systolic blood pressure be considered for cardiovascular risk evaluation: a study in middle-aged men and women J Am Coll Cardiol 2001 37 163 168 10.1016/S0735-1097(00)01092-5 11153732

[B53] Peralta CA Shlipak MG Wassel-Fyr C Bosworth H Hoffman B Martins S Association of antihypertensive therapy and diastolic hypotension in chronic kidney disease Hypertension 2007 50 474 480 10.1161/HYPERTENSIONAHA.107.088088 17664397

[B54] Iseki K Miyasato F Tokuyama K Nishime K Uehara H Shiohira Y Low diastolic blood pressure, hypoalbuminemia, and risk of death in a cohort of chronic hemodialysis patients Kidney Int 1997 51 1212 1217 10.1038/ki.1997.165 9083288

[B55] Franklin SS Khan SA Wong ND Larson MG Levy D Is pulse pressure useful in predicting risk for coronary heart Disease? The Framingham heart study Circulation 1999 100 354 360 10.1161/01.CIR.100.4.354 10421594

[B56] Arulkumaran N Diwakar R Tahir Z Mohamed M Kaski JC Banerjee D Pulse pressure and progression of chronic kidney disease J Nephrol 2010 23 189 193 20119928

[B57] Ramirez SP McClellan W Port FK Hsu SI Risk factors for proteinuria in a large, multiracial, southeast Asian population J Am Soc Nephrol 2002 13 1907 1917 10.1097/01.ASN.0000018406.20282.C8 12089388

[B58] Rema M Premkumar S Anitha B Deepa R Pradeepa R Mohan V Prevalence of diabetic retinopathy in urban India: the Chennai Urban Rural Epidemiology Study (CURES) eye study, I Invest Ophthalmol Vis Sci 2005 46 2328 2333 10.1167/iovs.05-0019 15980218

[B59] Mohan V Meera R Premalatha G Deepa R Miranda P Rema M Frequency of proteinuria in type 2 diabetes mellitus seen at a diabetes centre in southern India Postgrad Med J 2000 76 569 573 10.1136/pmj.76.899.569 10964123 PMC1741744

[B60] Wu Y Tai ES Heng D Tan CE Low LP Lee J Risk factors associated with hypertension awareness, treatment, and control in a multi-ethnic Asian population J Hypertens 2009 27 190 197 10.1097/HJH.0b013e328317c8c3 19145784

[B61] Sabanayagam C Shankar A Saw SM Tai ES Wong TY The association between socioeconomic status and overweight/obesity in a Malay population in Singapore Asia Pac J Public Health 2009 21 487 496 10.1177/1010539509343957 19661104

